# The sperm-interacting proteome in the bovine isthmus and ampulla during the periovulatory period

**DOI:** 10.1186/s40104-022-00811-2

**Published:** 2023-02-17

**Authors:** Coline Mahé, Régis Lavigne, Emmanuelle Com, Charles Pineau, Aleksandra Maria Zlotkowska, Guillaume Tsikis, Pascal Mermillod, Jennifer Schoen, Marie Saint-Dizier

**Affiliations:** 1 CNRS, IFCE, INRAE, Université de Tours, PRC, 37380 Nouzilly, France; 2 Univ Rennes, Inserm, EHESP, Irset (Institut de Recherche en Santé, Environnement Et Travail) - UMR-S 1085, F-35000 Rennes, France; 3 Univ Rennes, CNRS, Inserm, Biosit UAR 3480 US_S 018, Protim Core Facility, 35000 Rennes, France; 4 Institute of Reproductive Biology, Leibniz Institute for Farm Animal Biology, FBN, Dummerstorf, Germany; 5 Present Address: Department of Reproduction Biology, Leibniz Institute for Zoo and Wildlife Research (IZW), Berlin, Germany

**Keywords:** Ampulla, Fallopian tube, Interactome, Isthmus, Oviduct, Ovulation, Post-ovulatory, Pre-ovulatory, Proteomics, Spermatozoa

## Abstract

**Background:**

Spermatozoa interact with oviduct secretions before fertilization in vivo but the molecular players of this dialog and underlying dynamics remain largely unknown. Our objectives were to identify an exhaustive list of sperm-interacting proteins (SIPs) in the bovine oviduct fluid and to evaluate the impact of the oviduct anatomical region (isthmus vs. ampulla) and time relative to ovulation (pre-ovulatory vs. post-ovulatory) on SIPs number and abundance.

**Methods:**

Pools of oviduct fluid (OF) from the pre-ovulatory ampulla, pre-ovulatory isthmus, post-ovulatory ampulla, and post-ovulatory isthmus in the side of ovulation were collected from the slaughterhouse. Frozen-thawed bull sperm were incubated with OF or phosphate-buffered saline (control) for 60 min at 38.5 °C. After protein extraction and digestion, sperm and OF samples were analyzed by nanoLC-MS/MS and label-free protein quantification.

**Results:**

A quantitative comparison between proteins identified in sperm and OF samples (2333 and 2471 proteins, respectively) allowed for the identification of 245 SIPs. The highest number (187) were found in the pre-ovulatory isthmus, i.e., time and place of the sperm reservoir. In total, 41 SIPs (17%) were differentially abundant between stages in a given region or between regions at a given stage and 76 SIPs (31%) were identified in only one region × stage condition. Functional analysis of SIPs predicted roles in cell response to stress, regulation of cell motility, fertilization, and early embryo development.

**Conclusion:**

This study provides a comprehensive list of SIPs in the bovine oviduct and evidences dynamic spatio-temporal changes in sperm-oviduct interactions around ovulation time. Moreover, these data provide protein candidates to improve sperm conservation and in vitro fertilization media.

**Supplementary Information:**

The online version contains supplementary material available at 10.1186/s40104-022-00811-2.

## Background

Upon the end of their migration through the female genital tract, mammalian spermatozoa encounter secretions from the oviduct, where the final steps of sperm capacitation and fertilization take place [1]. Before ovulation, a subpopulation of sperm binds to oviduct epithelial cells in the isthmus, the caudal part of the oviduct, and builds the so-called functional sperm reservoir [2, 3]. Around the time of ovulation, spermatozoa are progressively released towards the ampulla, the distal part of the oviduct, where they meet the oocyte [1]. Numerous in vitro studies have demonstrated that oviduct fluid (OF) proteins modulate sperm viability and mobility in cattle [4–7] and humans [8]. Furthermore, the effect of OF proteins on sperm varies according to the estrous cycle stage and the oviduct region from which the OF originates [5, 6]. In particular, OF proteins from the peri-ovulatory (non-luteal) phase better favored post-thawed sperm viability, acrosome integrity, and motility compared to OF proteins from the luteal phase in cattle [6]. In addition, proteins from the isthmus preserved post-thawed sperm motility and acrosome integrity compared to proteins from the ampulla [5]. Therefore, the synchronization of events leading to the acquisition of sperm fertilizing ability may be monitored by spatiotemporal regulation of sperm interactions with OF proteins.

Proteins in the OF have different origins. The epithelium lining the oviductal lumen actively produces proteins secreted or released as cargo of oviduct extracellular vesicles (oEVs) [9–11]. Furthermore, OF proteins can originate from epithelial cell death (apoptosis and necrosis). Proteins originating from sub-epithelial structures reach the lumen via diffusion or other transport mechanisms [12]. The bovine OF proteome is highly regulated according to the estrous cycle stage [9, 13]. In addition, recent work from our laboratory showed that the oviduct anatomical region (isthmus vs. ampulla) also has a significant impact on the OF proteome [14].

However, the molecular mechanisms underlying the dynamic and close-up dialog between spermatozoa and OF proteins around ovulation time are still poorly understood.

The membrane of bull sperm is a complex mixture of phospholipids, glycans, and proteins originating from the seminal plasma [15, 16]. These are all potential binding partners for proteins in the OF. Blots of bull sperm membranes incubated with labeled OF showed sperm-interacting proteins (SIPs) ranging from around 20 to 140 kDa [17–19]. Immunodetection identified some SIPs including oviductin (OVGP1) [18], heat shock proteins (HSP) A5 (also known as glucose-regulated protein 78, GRP78), and HSPA8 [20, 21]. Recently, quantitative proteomic approaches have emerged as methods to explore SIPs more in-depth [22–24]. In our previous study using quantitative proteomics, the whole oviduct was used for OF collection, precluding any evaluation of the spatial regulation of SIPs. We hypothesize that the sperm-OF protein interaction varies in number and abundance between the ampulla and isthmus and also during ovulation, which would allow us to identify candidates involved in the formation of the sperm reservoir (pre-ovulatory isthmus). Thus, the objectives of this study were to identify an exhaustive list of SIPs in the bovine OF and to evaluate the impact of the oviduct anatomical region (isthmus vs. ampulla) and time relative to ovulation (pre-ovulatory (Pre-ov) vs. post-ovulatory (Post-ov)) on SIPs numbers and abundance using nanoLC-MS/MS analysis.

## Methods

### Collection of bovine oviduct fluid from the ampulla and isthmus

Bovine OF were collected from adult cyclic cows at a local slaughterhouse (40 min from the lab). Pairs of oviducts and ovaries were immediately placed on ice to stop muscular contractions and post-mortem modifications, transported to the laboratory, and classified according to ovarian morphology as previously described [25]. Only oviducts ipsilateral to ovulation at either pre-ovulatory or post-ovulatory stage of cycle were used. Pre-ovulatory ovaries contained one corpus albicans and one follicle of 11–20 mm in diameter (approximately days 19–21 of cycle). Post-ovulatory ovaries contained one early corpus luteum consisting of red, loosely organized tissue less than 1 cm in diameter (approximately days 1–4 of cycle). After removing the ovaries and surrounding tissues, the oviducts were cut at the ampulla-isthmus junction. Preliminary experiments by immunoblotting showed that a minimal concentration of 3 mg/mL of proteins in OF was needed to observe a sperm signal for myosin 9 (MYH9), a protein known to interact with bull sperm [22, 26], after incubation with OF. In addition, preliminary experiments showed that at least two isthmus samples, the narrowest part of the oviduct, were needed to reach a minimum of 3.5 mg/mL of proteins after flushing with 200 µL of phosphate-buffered saline (PBS). Therefore, on collection day, pools of isthmus and ampulla samples from 2 cows at the Pre-ov or Post-ov stage were formed and serially flushed with 200 µL of sterile protein-free PBS at 4 °C: one oviduct region was flushed with 200 µL of PBS and this fluid was re-used to flush the second oviduct region from the second cow. In the following, the term “OF” refers to one pool of OF flushes. All OF were kept on ice before two successive centrifugations (2000 × *g* then 12,000 × *g*, 4 °C, 10 min each) to eliminate cells and cellular debris. One aliquot was used for protein concentration measurement using the Uptima BC Assay kit (Interchim, Montluçon, France). Bovine serum albumin was used as a standard. Aliquots of flushes were stored at − 80 °C until used for sperm incubation and proteomic analysis. In total, four OF were collected per region × stage condition resulting in 16 OF (2 cows per OF). On the day of incubation, one OF per region × stage condition (Pre-ov isthmus, Pre-ov ampulla, Post-ov isthmus and Post-ov ampulla) was thawed on ice and the OF at 3.5 mg/mL was divided into two aliquots of 100 µL to assess (1) sperm-interacting proteins by proteomics and (2) sperm motility, membrane fluididity and integrity (see below).

### Assessment of sperm motility, membrane fluidity, acrosome reaction and viability

Bull semen was stored in a protein-free preservation medium (OptiXcell, IMV Technologies, L’Aigle, France) in liquid nitrogen. Assessment of sperm parameters was achieved at the start of incubation (T0) and after 1 and 3 h of incubation with OF. Sperm motility, progressive motility, amplitude of lateral head (ALH), linearity (LIN), average path velocity (VAP), curvilinear velocity (VCL) and straight line velocity (VSL) were evaluated on a Mackler chamber by computer-assisted sperm analysis (IVOS II, Animal Breeder software, version 1.8, Hamilton Thorne, Massachusetts, United States). To avoid sperm binding to the slide, 1% of bovine serum albumin was added to the PBS control samples. In parallell, aliquots of sperm were incubated 10 min at room temperature in 200 µL PBS with either (i) 7.5 µmol/L propidium iodide (PI, 81845, Sigma, Missouri, United States) and 1 µmol/L peanut agglutinin fluorescein (PNA, FL-1071, Vector laboratories, New Jersey, United States) to assess sperm membrane integrity and acrosome reaction, or (ii) 1.5 µmol/L DRAQ7 (D15106, Invitrogen, Massachussetts, United States) and 2.7 µmol/L Merocyanine 540 (323756, Sigma, Missouri, United States) to assess sperm membrane fluidity (lipid disorder). Sperm were next analyzed by flow cytometry (MACSQuant Analyzer 10, Miltenyi Biotec, Bergisch Gladbach, Germany). Propidium iodure (filter 1) and DRAQ7 (filter 2) fluorescence was collected through a 655–730 nm filter, PNA fluorescence through a 500–550 filter (filter 3) and merocyanine 540 fluorescence through a 565–605 filter (filter 4). Voltage settings were 410 V for forward scatter, 446 V for side scatter, 470 V for filter 1, 350 V for filter 2, 420 V for filter 3 and 400 V for filter 4. Data analysis was performed on at least 20,000 events with Flowjo software (version 10.4). Four replicates were carried out.

### Sperm incubation with ampullary and isthmic fluid and preparation of protein samples

**Fig. 1:**
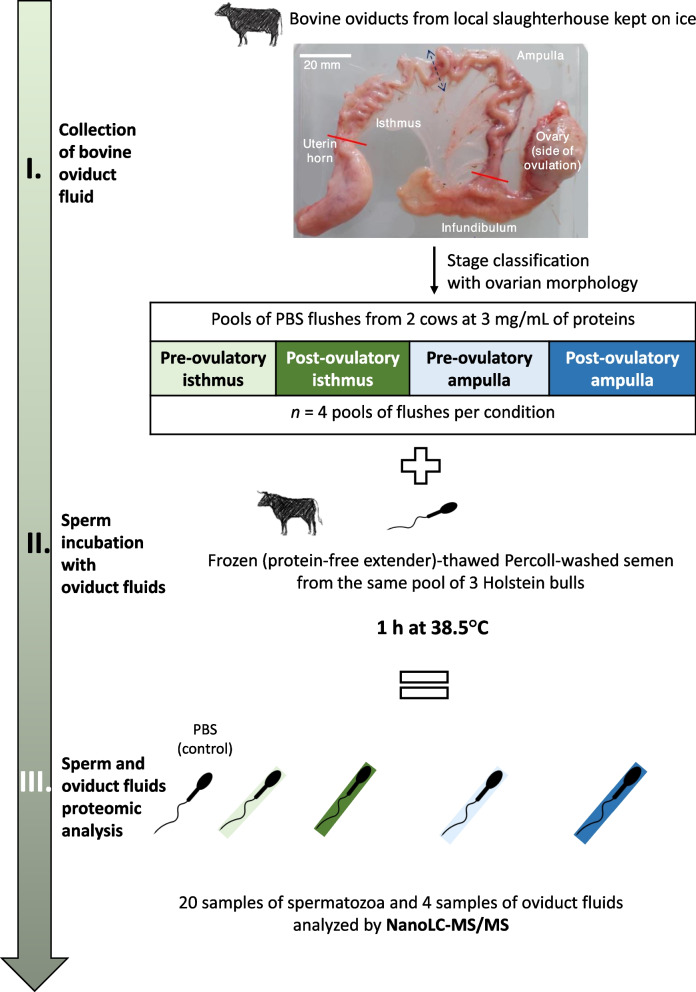
Experimental design for the identification of sperm-interacting proteome in the bovine oviduct fluid. PBS flushes from the pre-ovulatory isthmus, pre-ovulatory ampulla, post-ovulatory isthmus and post-ovulatory ampulla from two cows were pooled. Four pools of flushes per condition were constituted and incubated with the same pool of sperm for 1 h at 38.5 °C. Sperm and oviduct fluid were then analyzed by NanoLC-MS/MS

The experimental design of the study is shown in Fig. 1. In order to test the impact of female factors (oviduct region and ovulatory stage) on sperm-interacting proteins, the semen from the same pool of three Holstein bulls were used for all proteomic experiments. Sperm incubation with OF was undertaken as previously described [22] with slight modifications. A pool of 21 straws (7 straws per bull; approximately 20 × 10^6^ sperm per straw) were thawed in a water bath (37 °C, 3 min). The semen was washed on 45%–90% Percoll gradients (2.5 mL of Percoll 45 and 2.5 mL of Percoll 90; 700 g, 20 min, 25 °C; 1 mL of semen per gradient). The pooled sperm pellet was then washed with 2 mL of PBS (300 × *g*, 10 min, 25 °C) and sperm concentration in the pellet with some residual PBS was assessed in a Thoma cell counting chamber. For incubation with OF, 4.8 million of sperm in 20 µL PBS were added to 100 µL of PBS (control) or 100 µL OF containing 3.6 mg/mL protenis, resulting in a final sperm concentration of 40 × 10^6^/mL and OF concentration of 3 mg/mL proteins. Incubations were achieved in 1.5 mL polypropylene tubes for 60 min in a water bath at 38.5 °C. Preliminary data showed that this 60-min incubation time was enough to allow sperm-protein interactions without inducing capacitation, i.e., alteration of the sperm membrane [27]. After incubation, OF were recovered by centrifugation (4000 × *g*, 10 min, 4 °C), and sperm were washed twice in 1 mL of cold PBS (4000 × *g*, 10 min, 4 °C). Four replicates with four different pools of OF were done, resulting in 20 sperm samples.

Sperm pellets were resuspended in 25 μL of Tris–HCl at 10 mmol/L pH 6.8 containing 2% (w/v) of sodium dodecyl sulfate (SDS) and 0.05% (v/v) of a protease inhibitor cocktail (P2714, Sigma, Missouri, United States) then sonicated for 10 s at ambient temperature, and finally centrifuged for 10 min at 15,000 × *g* at 4 °C. The OF used for sperm incubation were pooled per region × stage condition, resulting in four pools of OF (Pre-ov isthmus, Pre-ov ampulla, Post-ov isthmus, and Post-ov ampulla). Samples of sperm and OF were assayed for protein concentration (Uptima BC Assay kit) and then stored at − 20 °C until proteomic analysis.

### Nanoliquid chromatography coupled with tandem mass spectrometry (NanoLC-MS/MS)

For proteomic analysis, sperm and OF pools (7 µg and 10 µg of proteins per sample, respectively) were prepared using the PreOmics iST-BCT kit (PreOmics GmbH, Planegg, Germany) following the manufacturer’s instructions. Briefly, proteins were lysed, denatured, reduced, and alkylated for 10 min at 95 °C, and then Trypsin/LysC digested for 60 min at 37 °C. Samples were then purified from salts, contaminants, and other detergents, using the provided spin cartridge (PreOmics GmbH). The resulting peptide mixtures were loaded on a 75 µm × 250 mm IonOpticks Aurora 2 C18 column (Ion Opticks Pty Ltd., Bundoora, Australia). A gradient of basic reversed-phase buffers (Buffer A: 0.1% formic acid, 98% H_2_O MilliQ, 2% acetonitrile; Buffer B: 0.1% formic acid, 100% acetonitrile) was run on a NanoElute HPLC System (Bruker Daltonik GmbH, Bremen, Germany) at a flow rate of 400 nL/min at 50 °C. The liquid chromatography (LC) run lasted for 120 min (2% to 15% of buffer B during 60 min; up to 25% at 90 min; up to 37% at 100 min; up to 95% at 110 min and finally 95% for 10 min to wash the column). The column was coupled online to a TIMS TOF Pro (Bruker Daltonik GmbH, Bremen, Germany) with a CaptiveSpray ion source (Bruker Daltonik). The temperature of the ion transfer capillary was set at 180 °C. Ions were accumulated for 114 ms, and mobility separation was achieved by ramping the entrance potential from − 160 V to − 20 V within 114 ms. The MS and MS/MS mass spectra were acquired with average resolutions of 60,000 and 50,000 full widths at half maximum (mass range 100–1700 *m/z*), respectively. To enable the PASEF method, precursor *m/z* and mobility information was first derived from full scan TIMS-MS experiments (with a mass range of *m/z* 100–1700). The quadrupole isolation width was set to 2 and 3 Th, and for fragmentation, the collision energies varied between 31 and 52 eV depending on the precursor mass and charge. TIMS, MS operation, and PASEF were controlled and synchronized using the control instrument software OtofControl 6.2 (Bruker Daltonik). LC–MS/MS data were acquired using the PASEF method with a total cycle time of 1.31 s, including 1 TIMS MS scan and 10 PASEF MS/MS scans. The 10 PASEF scans (100 ms each) contained, on average, 12 MS/MS scans per PASEF scan. Ion mobility-resolved mass spectra, nested ion mobility vs. *m/z* distributions, as well as summed fragment ion intensities were extracted from the raw data file with DataAnalysis 5.3 (Bruker Daltonik GmbH, Bremen, Germany). Signal-to-noise (S/N) ratios were increased by summations of individual TIMS scans. Mobility peak positions and peak half-widths were determined based on extracted ion mobilograms (± 0.05 Da) using the peak detection algorithm implemented in the DataAnalysis software. Feature detections were also performed using DataAnalysis 5.3 software and exported in .mgf format.

### Quantification and validation of proteins identified by mass spectrometry

Peptides were identified using the *Bos taurus* UniprotKB database (May 2021; 37,514 sequences) and the MASCOT software (version 2.5.1; Matrix Science, London, UK) using its automatic decoy database search to calculate a false discovery rate (FDR). The parameters used for database searches included trypsin as the enzyme (one missed cleavage allowed), carbamidomethylcysteine as a fixed modification, oxidation of methionine, and N-terminal protein acetylation as variable modifications. Monoisotopic mass was considered, and mass tolerance was set at 15 ppm for MS ions and 0.05 Da for MS/MS ions. Mascot results from the target and decoy databases were then incorporated into Scaffold Q + software (version 5.0.1, Proteome Software, Portland, USA). The peptide and protein identification thresholds were set to 95.0% as specified by the Peptide Prophet algorithm [28] and the Protein Prophet algorithm [29]. All proteins containing at least two unique peptides with an FDR < 0.01% were considered for protein quantification. Protein abundance was assessed using the Spectrum Count quantitative method and the “weighted spectra” option of the Scaffold software, as previously described [14]. The weighted spectra option of Scaffold is based on the assignment of each peptide to a weight according to whether they are shared or not between identified proteins. The normalization of data was achieved by multiplying each spectrum count in each sample by the average count over the sample’s total spectrum count.

### Identification of sperm-interacting proteins

Proteins were considered as SIPs originating from the OF if they met the following criteria: (i) detection by nanoLC-MS/MS in the OF with a minimum level of 5.0 NWS and (ii) detected exclusively in sperm treated with OF (i.e., no detection in control sperm incubated with PBS) with ≥ 5.0 NWS on average in at least one stage × region condition or (iii) detected in significantly higher abundance in OF-treated sperm than in controls (*t*-test with a *P*-value ≤ 0.05) with at least 5.0 NWS and a mean treated:control fold-change ratio ≥ 3.0 in OF-treated condition.

### Immunodetection of HSPA5, MYH9, and PRDX6 in protein samples from oviduct fluid and spermatozoa

The SIPs HSPA5, previously known to interact with bull sperm [20, 21], MYH9, and PRDX6 were validated by immunoblotting. The semen from three Holstein bulls different than those used for proteomics and stored under the same conditions (OptiXcell preservation medium, liquid nitrogen) were incubated with OF from ampulla or isthmus at Pre-ov and Post-ov as described earlier. PBS was used as control. After sperm protein extraction, 5 µg of OF and 20 µg of sperm proteins were migrated on a 4%–20% SDS-PAGE gel (Bio-Rad, Hercules, CA, USA). Semi-liquid transfers on a nitrocellulose membrane were performed using the Trans-blot Turbo Transfer System (Bio-Rad) and the “Mixed molecular weight” program. Membranes were stained for 5 min with Ponceau red to check the homogeneity between lanes and for data normalization [30]. Proteins were then blocked for 2 h with Tris-buffered saline supplemented with 0.5% Tween 20 supplemented with 5% of lyophilized low-fat milk (TBST–milk). Membranes were then incubated with primary antibodies (see the list of primary antibodies in Additional file 1: Table S1), diluted in TBST-milk overnight at 4 °C, and washed (× 4, 10 min) with TBST. Membranes were incubated for 1 h at 37 °C with secondary antibodies conjugated to horseradish peroxidase diluted in 1:2500 in TBST-milk (Sigma, Missouri, United States) (see the list of secondary antibodies in Additional file 1: Table S1). The peroxidase was revealed with chemiluminescent substrates (ECL Clarity Max, Bio-Rad) and the images were digitized with a cooled CCD Camera (ImageMaster VDS-CL, Amersham Biosciences). Signal quantification was assessed by densitometry using an Image Scanner (Amersham Biosciences, GE Healthcare Life Sciences) and the TotalLab Quant software (version 11.4, TotalLab, Newcastle upon Tyne, UK). Three replicates with different OF were performed for each protein.

### Statistical analysis of data

Data were analyzed using the Rstudio software (version 1.4.1106). CASA and flow cytometry data were analyzed by analysis of variance (ANOVA) and Tuckey post test at each time (*P*-value ≤ 0.05). Pairwise comparisons of SIPs between regions at a given stage and between stages at a given region were analyzed by *t*-tests. Proteins were considered differentially abundant with a *P*-value ≤ 0.050. The distribution of SIPs between conditions was analyzed by Venn diagrams using jvenn tool online [31].

### Prediction of secretory pathways and functional enrichment analysis

In order to detect a signal peptide or to predict unconventional secretion pathways, FASTA sequences of SIPs were used as input into the Outcyte online tool (version 1.0) [32]. The presence of signal peptides was further confirmed by SignalP 6.0 in Eukarya organism [33]. In order to predict a secretion via extracellular vesicles (EVs), the gene list of SIPs was compared with gene lists previously reported in bovine [9], feline [34], and porcine [35] oviduct EVs. Functional classification terms for gene ontology (GO), molecular function (MF), and biological processes (BP) were obtained using the Protein Analysis Through Evolutionary Relationships (PANTHER version 17.0) with the gene list as input and *Bos taurus* as background. Enrichment analysis for GO, MF, and BP were carried out using the SIP gene lists as input in Metascape and the *Homo Sapiens* database as background [36]. To go further into the potential functions of SIPs, membership annotations (in GO, MF, and BP name or description) for “cell motility,” “sperm,” “fertilization,” and “oviduct” were also analyzed with Metascape.

Protein–protein interaction networks and KEGG pathway enrichment analysis (*P*-value < 0.01) of SIPs were carried out using the corresponding list of gene names in STRING (Search Tool for the Retrieval of Interacting Genes/proteins; version 11.5) and *Bos taurus* as background [37]. For the construction of the interaction network, a minimum interaction score of 0.4 and the following interaction sources (text mining, experiments, databases, and co-expression) were selected in the settings. Line thickness between proteins represents the degree of confidence of interaction predictions. The disconnected nodes in the network were discarded. The networks were then imported into Cytoscape for color adjustments (version 3.9.0) [38].

## Results

### Sperm parameters during incubation with oviduct fluid

**Fig. 2:**
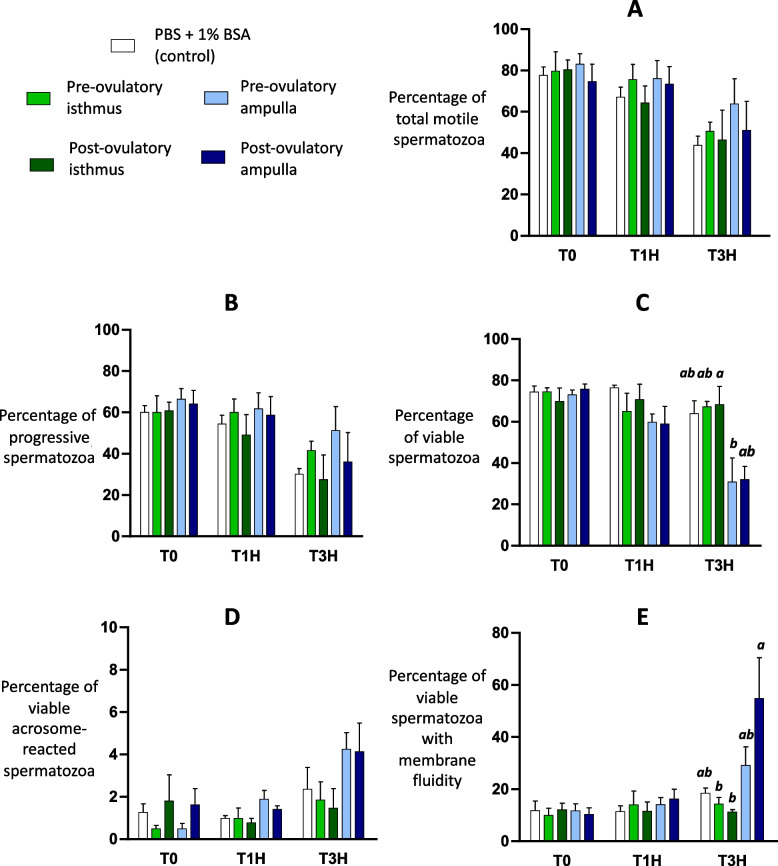
Sperm parameters during incubation with oviduct fluid (treated groups) or PBS (controls). Sperm motility (**A**, **B**) was assessed by computer assisted sperm analysis (CASA). Sperm viability (**C**), acrosome reaction (**D**) and membrane lipid disorder (**E**) were assessed by flow cytometry. Data are means ± SEM of 4 replicates

After 1 h of incubation with OF, no difference between conditions were found for sperm motility parameters, membrane fluidity and acrosome reaction (Fig. 2 and Additional File 7: Fig. S1). After 3 h, the OF from post-ovulatory ampulla increased sperm membrane fluidity compared to that from isthmus (*P* < 0.05). Furthermore, the OF from post-ovulatory isthmus maintained higher sperm viability compared to that from pre-ovulatory ampulla (*P* < 0.05).

### Classification and secretory pathways of identified sperm-interacting proteins

The sperm-interacting proteome was assessed after 1 h of incubation with OF in all conditions.

**Fig. 3:**
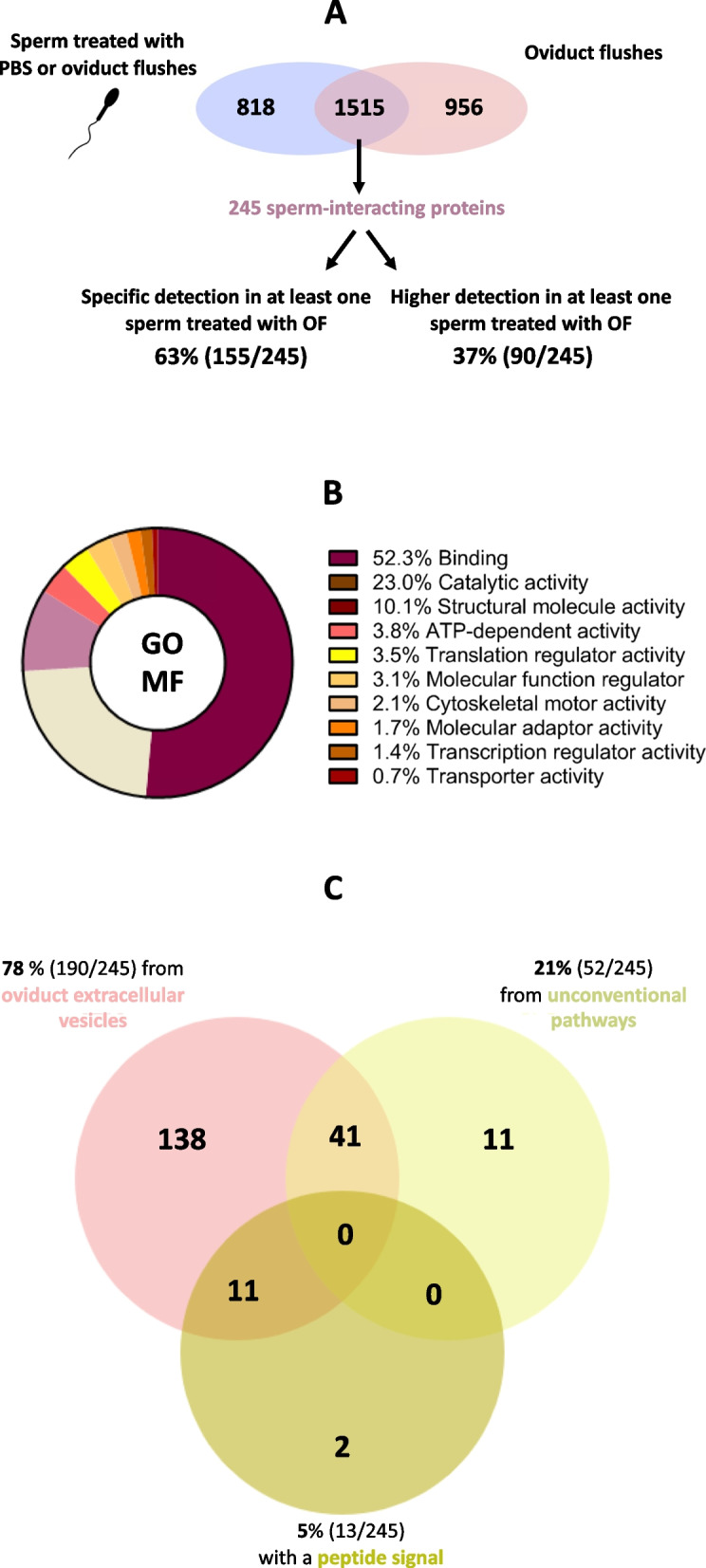
Classification of sperm-interacting proteins according to their (**A**) criteria of identification, (**B**) gene ontology (GO) molecular functions (MF) from PANTHER, and (**C**) predicted secretory pathways. Prediction of secretion by unconventional pathways and with a peptide signal were carried out using Outcyte and SignalP 6.0 tools online. The proportion of SIPs potentially secreted by oviduct extracellular vesicles were previously reported in cattle [9], cats [34], and pigs [35]

A total of 2333 proteins were identified in sperm treated with OF, of which 245 (11%) were defined as SIPs originating from the OF. The 245 SIPs represented 10% of the 2471 proteins quantified with more than 5.0 normalized weighted spectra (NWS) in the different pools of OF and 18.9% of the 1294 proteins shared by sperm and OF (Fig. 3A). Table 1 shows the top 25 SIPs when considering the four stage × region conditions (see all proteins identified in Additional file 2: Table S2 and all SIPs with quantitative data in Additional file 3: Table S3). Myosin heavy chain 9 (MYH9) was the most abundant SIP in all conditions.

**Table 1:** Top 25 most abundant sperm-interacting proteins (SIPs) identified in the oviduct fluid (OF) derived from the ampulla and isthmus just before (Pre-ov) and after (Post-ov) ovulation

			OF:control sperm ratio in					
Protein name	Gene symbol	Mean NWS in OF-treated sperm	Pre-ov isthmus	Pre-ov ampulla	Post-ov isthmus	Post-ov ampulla	Predicted secretion pathways	Reported role in sperm physiology and fertilization
Myosin heavy chain 9	*MYH9*	284.4	45.9	66.4	54.3	119.4	EVs	OVGP1 partner on human sperm [26]
Myosin heavy chain 14	*MYH14*	184.0	66.4	75.3	86.2	140.7	EVs	-
AHNAK nucleoprotein	*AHNAK*	101.4	512.7	341.1	509.2	287.5	EVs	-
Endoplasmic reticulum chaperone BiP	*HSPA5*	83.8	3.3	3.0	3.5	4.5	EVs and classical	Human sperm capacitation [39] and binding to zona pellucida [40]
Tubulin polymerization-promoting protein family member 3	*TPPP3*	81.6	17.8	17.4	17.9	7.2	EVs	-
Myosin-10	*MYH10*	70.3	*No detection in control*				EVs	-
Heat shock protein beta-1	*HSPB1*	59.9	134.9	284.1	199.6	179.0	EVs	-
Myosin light polypeptide 6	*MYL6*	58.1	22.3	31.5	33.9	34.0	EVs and unconv	-
Oviduct-specific glycoprotein	*OVGP1*	52.5	28.7	24.5	21.3	22.1	EVs	Bovine sperm capacitation [41], binding to zona pellucida and monospermy [42]
Heat shock cognate 71 kDa protein	*HSPA8*	38.6	3.0	3.3	2.7	2.5	EVs	Bovine sperm survival, membrane fluidity and monospermy [21]
Tropomodulin 3	*TMOD3*	36.0	23.9	50.2	44.0	34.7	Unconv	-
Protein disulfide-isomerase	*PDIA3*	33.7	4.7	4.2	5.3	3.4	EVs and classical	Human sperm binding to zona pellucida [43]
Keratin, type II cytoskeletal 8	*KRT8*	32.7	332.5	129.3	338.4	66.3	EVs	-
Actin, cytoplasmic 1	*ACTB*	31.7	2.9	4.7	3.9	4.1	EVs	-
Actin, cytoplasmic 2	*ACTG1*	31.5	2.9	4.5	3.7	3.9	EVs	-
Endoplasmin	*HSP90B1*	28.9	4.2	4.1	3.4	5.3	EVs and classical	KO mouse unable to fertilize oocytes [44] Binding partner of PDIA4 for monospermy in pigs [45]
Hypoxia up-regulated 1	*HYOU1*	27.5	5.6	3.2	5.3	4.7	EVs	-
Elongation factor 1-alpha 1	*EEF1A1*	23.4	6.9	13.4	11.5	8.1	EVs and unconv	-
Tropomyosin alpha-3 chain	*TPM3*	23.3	*No detection in control*				EVs and unconv	-
Brain acid soluble protein 1	*BASP1*	22.9	5.1	8.9	4.1	4.5	EVs	-
Annexin A1	*ANXA1*	22.8	2.1	3.0	1.8	2.8	EVs and unconv	Bovine sperm binding to oviduct epithelial cells [46]
Na(+)/H(+) exchange regulatory cofactor NHE-RF1	*SLC9A3R1*	21.6	*No detection in control*				EVs	-
Protein disulfide-isomerase A4	*PDIA4*	20.7	26.8	26.8	27.3	31.7	EVs and classical	Penetration rate and monospermy in pigs [47] Binding partner of HSP90B1 for monospermy in pigs [45]
Annexin A2	*ANXA2*	20.6	69.6	118.3	69.2	78.9	EVs and unconv	Bovine sperm binding to oviduct epithelial cells [46]
40S ribosomal protein S3a	*RPS3A*	19.8	74.4	77.3	70.2	29.7	EVs and unconv	-

Proteins are classified in decreasing order of quantified level in OF-treated spermatozoa (mean ± SEM of 4 conditions; *n* = 4 replicates per condition). Fold-change ratios between OF-treated and control sperm groups are indicated for each condition (a value in italics means exclusive detection in OF-treated spermatozoa). The secretion pathway was predicted using Outcyte and SignalP and by comparison with reported lists of proteins in OF-derived EVs. ‘Classical’ means secretion via a signal peptide; ‘Unconv’ means via unconventional pathways (including paracrine and EV-secretion pathways); ‘EVs’ means previously reported in OF-derived EVs. The main roles reported in sperm physiology and/or fertilization in mammals are indicated (a dash means no report found).

*NWS*: normalized weighted spectra

Overall, 155 SIPs (63%), including MYH10 and tropomyosin alpha-3 chain (TPM3), were detected exclusively in at least one OF-treated sperm condition (with no detection in control samples). Furthermore, 90 SIPs (37%), including OVGP1, MYH9, HSPA5, and peroxiredoxin-6 (PRDX6), were detected at significantly higher abundances in at least one OF-treated sperm condition compared to the controls (*P*-value ≤ 0.05 and fold-change ratio ≥ 3.0; Table 1). Immunoblotting of sperm protein extracts showed higher signals in OF-treated than control sperm for HSPA5, and signals exclusively in OF-treated samples for MYH9 and PRDX6 (Additional file 8: Fig. S2).

The GO classification of SIPs using PANTHER indicated that 52% were binding proteins, 23% catalytic proteins, and 10% structural proteins (Fig. 3B). The prediction of protein secretory pathways indicated that 5% (13/245) of SIPs possessed a peptide signal and were predicted to be classically secreted, while 21% (52/245) were secreted via unconventional pathways. In addition, 78% (190/245) of SIPs were previously reported in oviduct extracellular vesicles in mammals [9, 34, 35] with overlaps with the two previous categories of secretion (Fig. 3C and Additional file 3: Table S3). Overall, 83% (203/245) of SIPs were predicted to be secreted by conventional or unconventional pathways.

### Impact of the oviduct anatomical region and peri-ovulatory stage on numbers and abundance of sperm-interacting proteins

**Fig. 4:**
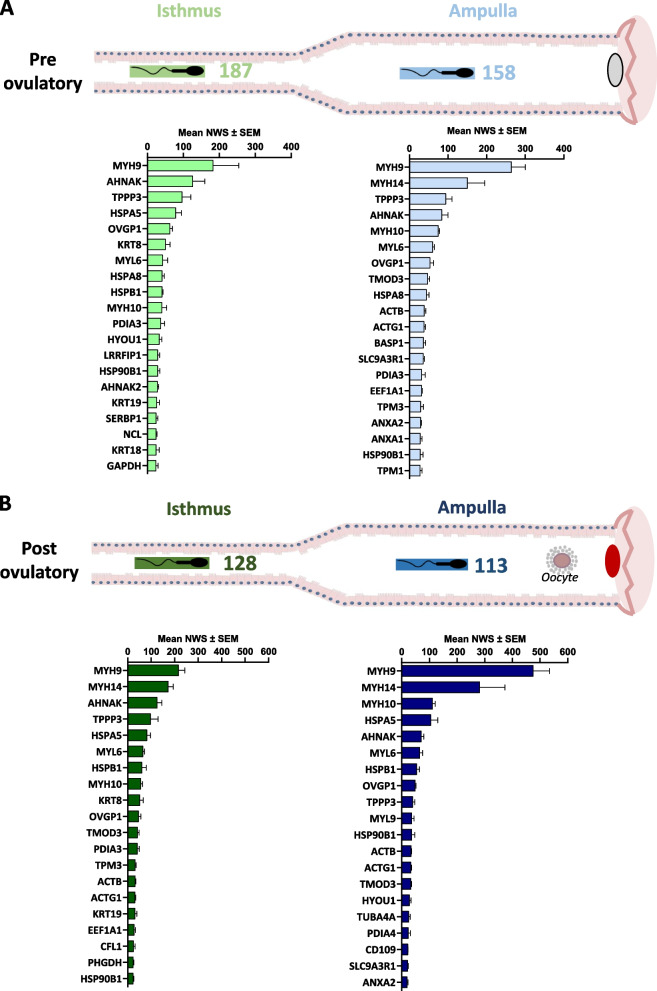
Numbers and mean normalized quantitative values (± SEM) of top 20 sperm-interacting proteins in the isthmus and ampulla at (**A**) pre-ovulatory and (**B**) post-ovulatory cycle stages

Figure 4A and B show counts and the top 20 most abundant SIPs in both oviduct regions at Pre-ov and Post-ov, respectively (see Additional file 4: Table S4 for quantitative values).

**Fig. 5:**
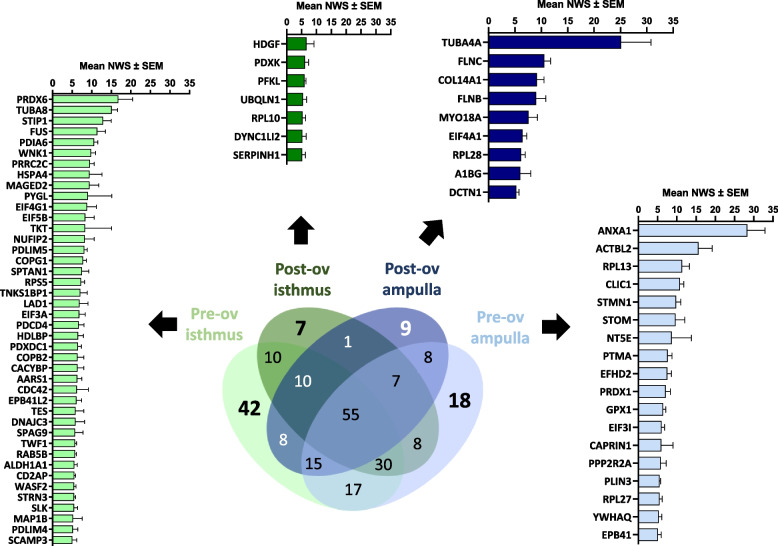
Distribution and mean normalized quantitative values (± SEM) of sperm-interacting proteins identified in only one oviduct region (isthmus versus ampulla) at pre-ovulatory and post-ovulatory cycle stages

The highest number of SIPs (187/245) was identified in the Pre-ov isthmus, i.e., time and place of oviduct reservoir. In contrast, the lowest (113/245) was identified in the Post-ov ampulla, i.e., time and place of fertilization. This difference in numbers of SIPs could not be explained by differences in protein numbers in the OF (Additional file 2: Table S2). Furthermore, 76 SIPs (31%) were identified in only one region × stage condition: 42 (17%) in the Pre-ov isthmus, 18 (7%) in the Pre-ov ampulla, 9 (4%) in the Post-ov ampulla, and 7 (3%) in the Post-ov isthmus (Fig. 5).

**Fig. 6:**
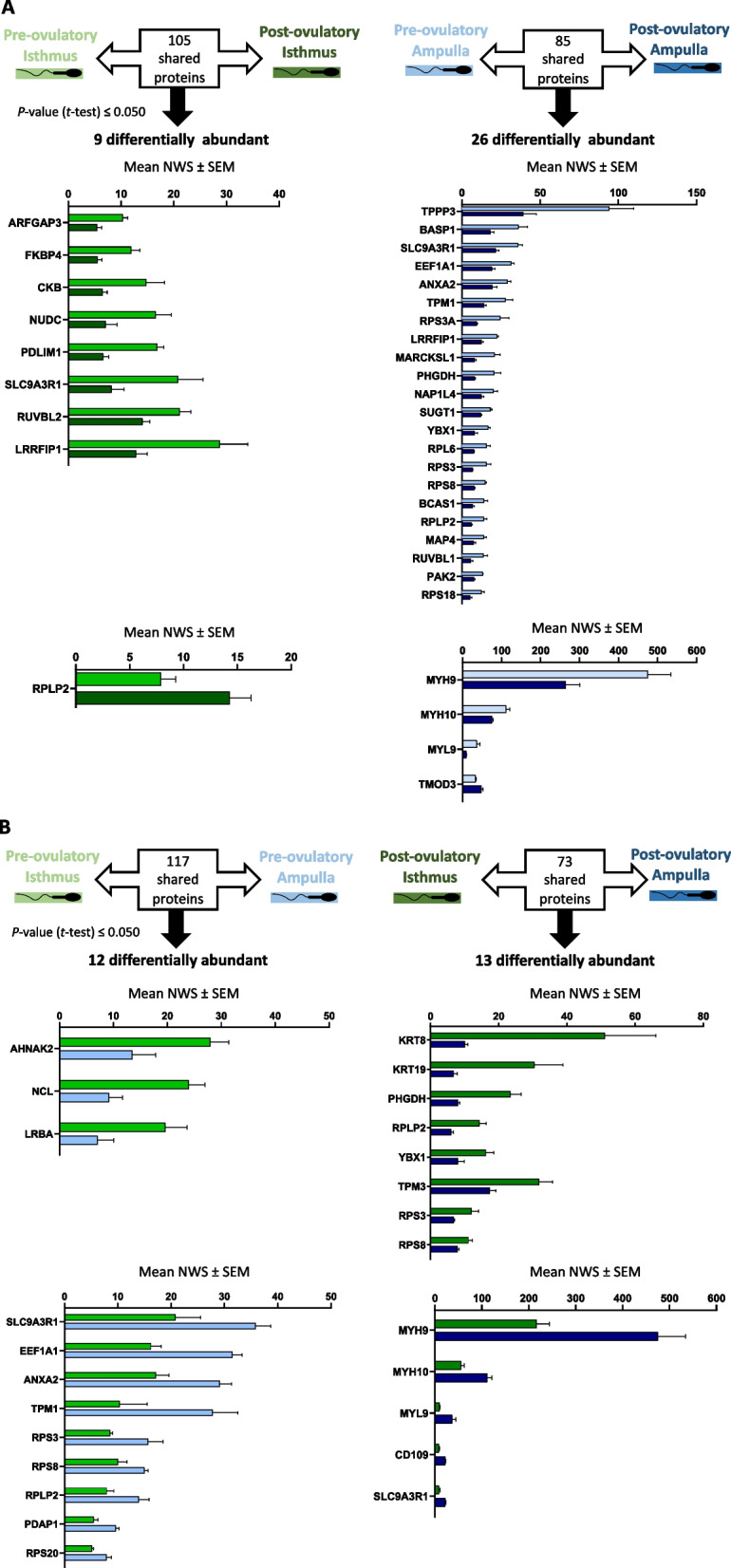
Numbers and mean normalized quantitative values (± SEM) of differentially abundant sperm-interacting proteins (**A**) between pre-ovulatory and post-ovulatory cycle stages in the isthmus and ampulla and (**B**) between ampulla and isthmus at pre-ovulatory and post-ovulatory stages. SIPs shared between either stages or regions were considered as differentially abundant with a *P*-value ≤ 0.050 after the Student’s *t*-test

In total, the abundance of 41 SIPs (17%) was impacted by the peri-ovulatory stage or anatomical region (Fig. 6). Considering the SIPs common to both stages in a given region, 9% (9/105) and 31% (26/85) were differentially abundant between Pre-ov and Post-ov in the isthmus and ampulla, respectively (Fig. 6A). Considering the SIPs common to both regions at a given stage, 10% (12/117) and 18% (13/73) were differentially abundant between isthmus and ampulla at Pre-ov and Post-ov, respectively (Fig. 6B). Furthermore, there was no correlation between the initial abundance of SIPs in the OF and their abundance in OF-treated spermatozoa (see scatter plots in Additional file 9: Fig. S3).

### Functional analysis of SIPs and protein–protein interaction networks

**Fig. 7:**
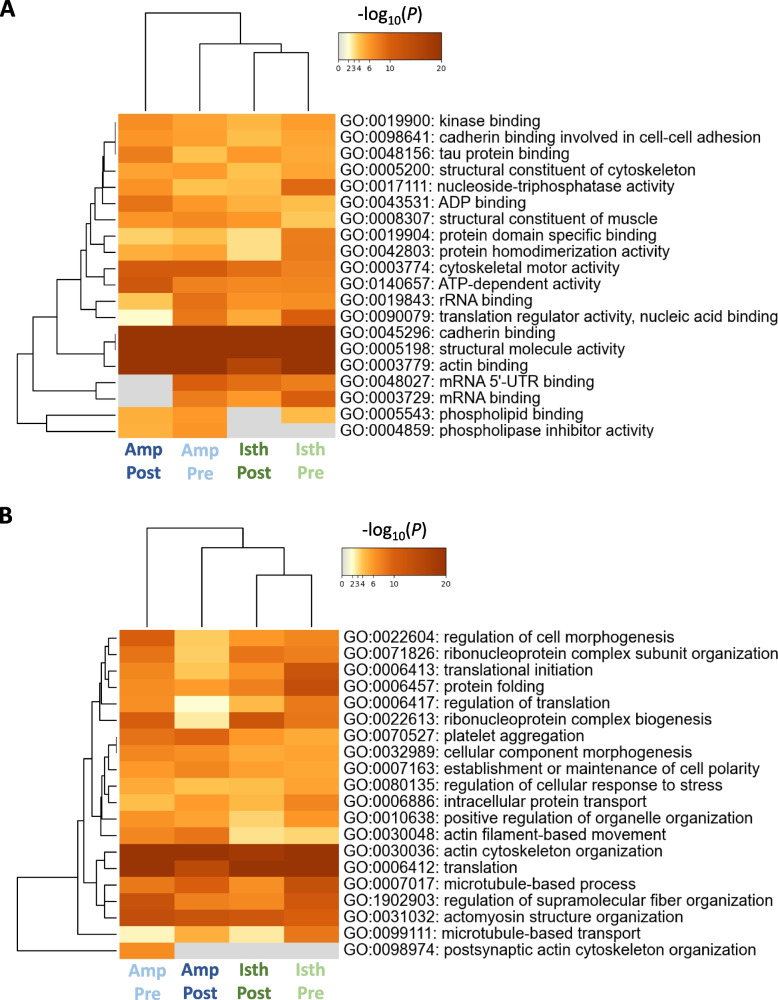
Functional enrichment analysis of sperm-interacting proteins for Gene ontology (**A**) Molecular Functions and (**B**) Biological Processes in the ampulla and isthmus pre-ovulatory and post-ovulatory stages. The analysis was made using Metascape**.** Amp Post: post-ovulatory ampulla; Amp Pre: pre-ovulatory ampulla; Isth Post: post-ovulatory isthmus; Isth Pre: pre-ovulatory isthmus

The top 20 GO terms of the Metascape enrichment analysis for molecular functions (GO MF) and biological processes (GO BP) of SIPs are presented in Fig. 7A and B, respectively (see all GO terms and *P*-values in Additional file 5: Table S5). A significant overrepresentation of SIPs in functions such as cadherin binding, structural molecule activity, actin binding, cytoskeletal motor activity, and ATP-dependent activity was seen. Furthermore, SIPs were enriched in processes like cytoskeleton organization, microtubule-based processes, actomyosin structure organization, and regulation of cellular responses to stress.

**Fig. 8:**
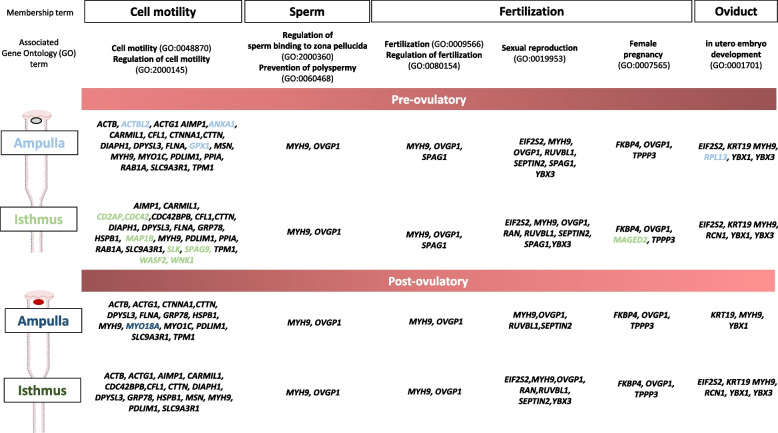
Sperm-interacting proteins associated with terms related to cell motility, sperm, fertilization, and oviduct. Membership analysis was carried out with the online Metascape tool and the *Homo Sapiens* database. For each membership term, the gene ontology (GO) terms associated with SIPs are indicated below. The colored gene symbols indicate SIPs identified in only one region × stage

The possible roles of SIPs in periconception events in the oviduct were further studied using the Metascape membership tool [36]. Proteins associated with the membership terms ‘cell motility,’ ‘sperm,’ ‘fertilization,’ and ‘oviduct’ are presented in Fig. 8. In total, 46 SIPs were retrieved with these particular terms, including 32 in cell motility, 11 in fertilization, and 7 in oviduct functions. Reported roles in sperm physiology and fertilization are also indicated in Table 1.

**Fig. 9:**
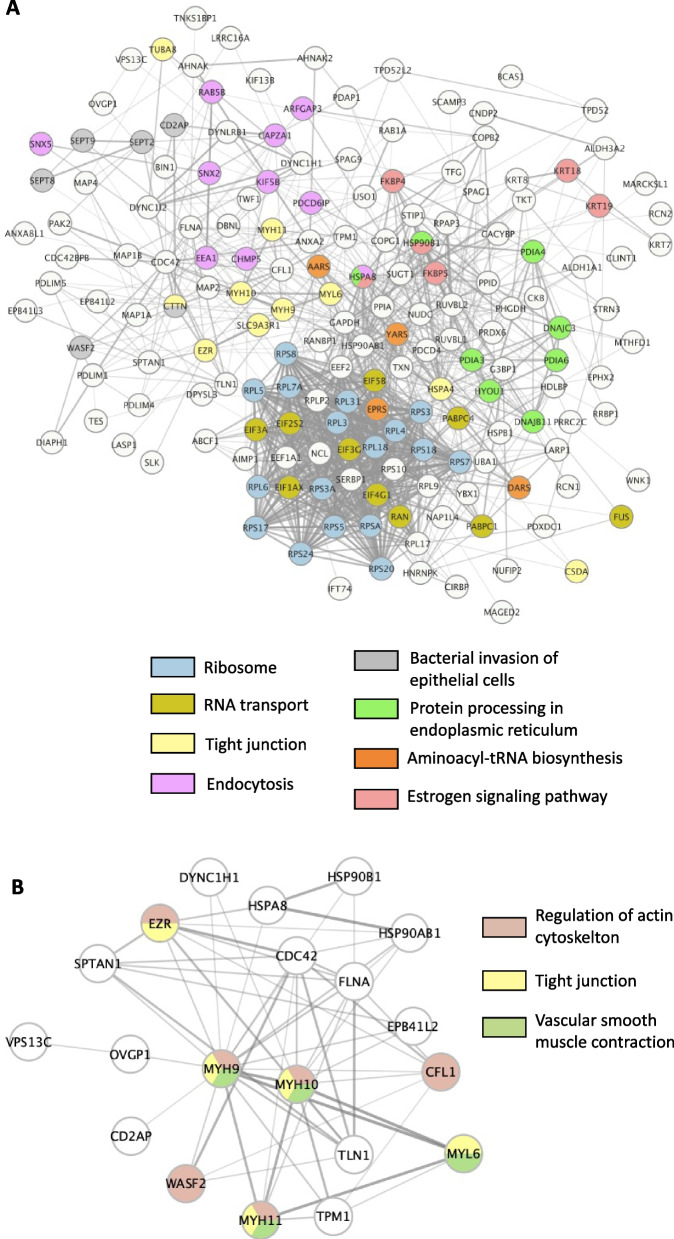
Predicted protein–protein interaction networks and KEGG pathway enrichment analysis of (**A**) all sperm-interacting proteins in the pre-ovulatory isthmus or (**B**) MYH9 and OVGP1. Networks were built using STRING and Cytoscape with a minimum interaction score of 0.4. Line thickness between proteins represents the degree of confidence prediction of the interaction

To analyze the possible interactions between SIPs, protein–protein interaction networks were built separately in each condition using STRING. Figure 9A shows the network obtained in the Pre-ov isthmus for all SIPs. Numerous interactions were described between SIPs, some of them being involved in pathways related to RNA and protein processing, tight junction and estrogen signaling. As MYH9 was the most abundant SIP, a focus on this protein is shown in Fig. 9B (see Additional file 10: Fig. S4 for other conditions and Additional file 6: Table S6 for network details). In this network, MYH9 was in a node core interacting with 19 other SIPs, including OVGP1, MYH10, HSP90B1, and HSPA8. MYH9 and other myosins (MYH10, MYH11) were involved in pathways related to regulation of actin cytoskeleton, tight junction and vascular smooth muscle contraction.

## Discussion

Using an MS-based quantitative approach, this study provides a comprehensive list of SIPs in the bovine OF, including proteins already well known to interact with bull spermatozoa. These data show changes induced by the oviduct anatomical region and ovulation time on the number and abundance of SIPs, which correlates with the hypothesis that synchronization of events leading to fertilization is monitored by dynamic interactions between sperm and oviduct secretions.

### Identification of sperm-interacting proteins by quantitative proteomics

Overall, 11% of proteins detected in sperm treated with OF were considered to be SIPs. In a previous proteomic study in pigs, 63% (64/102) of sperm proteins were considered as SIPs after incubation with OF [24]. However, in that study, all proteins shared between OF-treated sperm and OF were classified as potential SIPs without comparison with a sperm control group and quantitative criteria [24]. Here we used quantitative proteomics and comparison between normalized levels of proteins in the OF and spermatozoa incubated with or without OF for identifying SIPs. Two criteria were required to define a SIP: significant detection in the OF used for sperm incubation and exclusive or significantly higher abundance in OF-treated sperm with a fold-change ratio of at least 3.0 compared to controls. Of note, the proteins well known to interact with bull sperm, including HSPA8 [21], HSPA5 (GRP78) [20], and OVGP1 [18], were all quantified with a higher abundance in OF-treated than control sperm, confirming the interest of using quantitative proteomic approaches to indentify sperm-interacting proteins. In our previous proteomic study, HSPA8, HSPA5 and OVGP1 were detected in OF-treated sperm but not in control sperm [22]. This difference is likely due to higher peptide separation by chromatography and greater sensitivity of the MS instrument used, which led to more proteins identified in all groups of spermatozoa (around 2300 vs. 500 proteins in our previous study). Furthermore, MYH9 and PRDX6 were detected higher in OF-treated sperm than in PBS by proteomics whereas they were exclusively detected in OF-treated sperm by immunoblotting, which may be explained by the different sensitivities of the methods.

### Origin and possible binding mechanisms of sperm-interacting proteins

As expected, a majority (52%) of SIPs were classified by PANTHER as binding proteins. Note that OF were collected by gentle pressure to avoid cell lysis, then two successive centrifugations were carried out to eliminate cells and cellular debris. Thus, the SIPs were present in the oviduct lumen exclusively. The SIPs overrepresented in actin cytoskeleton organization processes included several myosins (MYH9, MYH10, MYH14, and MYL6), tropomyosin 3 (TPM3), and some heat shock proteins (HSP90B1, HSPA8, HSPA5). Consistent with our data, a previous study that used proteomics to identify a subset of 70 kDa oviductal surface proteins that bound to bull spermatozoa reported HSPA8, HSP90B1, HSPA5, and TPM3 as potential SIPs [21].

To our knowledge, receptors for the above proteins on sperm membranes are not known. However, SIPs may anchor to the sperm membrane through lipid interactions. MYH9, MYH10, and MYH14 belong to the class II of mammalian non-muscle myosins, consisting of two heavy chains, two essential light chains, and a pair of regulatory light chains [48]. MYH9, MYH10, and MYH14 (also known as non-muscle myosins II-A, II-B, and II-C) were reported to bind to liposomes made with various phospholipids with concomitant dissociation of their regulatory chain [49].

The SIPs may also interact through binding to sperm surface proteins. MYH9 has been previously identified as a binding partner of OVGP1 on the surface of human spermatozoa [26].

In addition, SIPs may interact with sperm through rapid fusion or binding with oviduct EVs. Extracellular vesicles are important mediators of cell–cell communications as they transfer not only proteins but also small RNAs, phospholipids, and metabolites [50]. Of the 245 SIPs, 78% were previously reported in oviductal EVs, including exosomes and microvesicles. This proportion was much higher than those reported for proteins identified in the whole bovine OF [14], indicating an enrichment in EV-secreted proteins among SIPs. Interactions between spermatozoa and oviduct EVs have already been described in cats [34], cattle [10], pigs [51], and mice [52]. In mice, it was shown that the plasma membrane calcium/calmodulin-dependent calcium ATPases 4, which has an essential function in sperm motility, was delivered to sperm through membrane fusion with oviduct EVs via integrins and CD9 tetraspanin [52]. In cattle, spermatozoa have been shown to uptake EVs derived from bovine OF after only 15 min of co-incubation. Those interactions regulated protein tyrosine phosphorylation and intracellular calcium levels, two processes related to sperm capacitation [10]. In addition, EVs derived from follicular fluid were reported to modulate bull sperm viability, capacitation, and acrosome reaction [53]. Taken together, these data reinforce the idea that oviduct EVs may be key players of protein cargo delivery for the acquisition of sperm fertilizing ability in vivo.

### Spatiotemporal regulation of sperm-interacting proteins

Up to 31% of SIPs changed in numbers and abundance between oviduct anatomical regions and peri-ovulatory stages. This is in line with our previous report showing 9 differentially abundant SIPs over the 14 identified in luteal, pre-ovulatory, and post-ovulatory OF [22]. Also consistent with our results, the effects of OF and OF-derived EVs on bull sperm, as reported by several in vitro studies, change according to the anatomical region [10, 54] and cycle stage [27].

There was no relationship between the initial abundance of SIPs in the OF and their abundance in OF-treated spermatozoa. This indicates highly specific sperm-protein interactions.

In order to avoid a bull effect in this study, the same pool of semen from three bulls was used for all incubations with OF. Therefore, the changes observed in sperm proteomics are expected to be due to differences in OF protein composition. It has been reported that up to 12% of proteins in the OF change in abundance according to the anatomical region and peri-ovulatory stage [14]. As shown by the protein–protein interaction network built by STRING, most SIPs may interact with each other. Therefore, some SIPs may anchor rapidly to the sperm membrane and lead to other protein interactions over time and depending on the OF protein composition. Our data suggest dynamic sperm-OF interactions as the sperm progresses toward the oocyte. The beneficial role of sequential interactions on sperm fertilizing ability remains to be tested.

### Sperm-interacting proteins in the pre-ovulatory isthmus and possible impact on sperm binding to the sperm reservoir

The pre-ovulatory isthmus is the expected place of sperm storage in mammals [3, 55, 56]. A total of 42 SIPs were identified exclusively in the isthmus just before ovulation, including peroxiredoxin-6 (PRDX6). PRDX6 is well known to play roles in protecting cells from oxidative stress [57–59] and was reported to protect human and mouse spermatozoa from oxidation [60]. In particular, PRDX6 has both peroxidase and calcium-independent phospholipase activites. Inhibition of PRDX6 increased sperm DNA damage [58] and the production of reactive oxygen species during capacitation [59].

Sperm binding to oviduct epithelial cells is mediated by membrane glycoproteins, including annexins A1, A2, A4, and A5 [46], HSPA5 [20], and HSPA8 [21]. ANXA2, HSPA5, and HSPA8 were among the most abundant SIPs in the Pre-ov isthmus. Furthermore, HSPA5, ANXA1, and ANXA2 were among the top 20 most abundant SIPs in the Pre-ov ampulla, while ANXA4 and ANXA5 were identified as SIPs in the Pre-ov and Post-ov ampullas. Therefore, a binding competition between the OF and sperm receptors on luminal oviduct cells is expected, which may lead to a decrease in sperm binding to the sperm reservoir. Further studies are needed to clarify the impact of SIPs on sperm binding to oviduct epithelial cells.

### Potential roles of SIPs on sperm capacitation and focus on OVGP1 and MYH9

The reported roles of the most abundant SIPs in sperm physiology and fertilization are indicated in Table 1. Sperm capacitation is a timed process starting with an elevation of intracellular calcium and includes phospholipid remodeling, protein tyrosine phosphorylation, and hyperactivated motility, allowing sperm to cross the cumulus cells and the zona pellucida and eventually fuse with the oocyte [61]. After 3 h of co-incubation, OF from the ampulla increased sperm membrane fluidity compared to OF from the isthmus. In accordance, proteins [5] and oEVs [10] purified from the ampullary OF induced the capacitation of bull sperm in vitro. Though the contribution of SIPs to the acquisition of hyperactivated motility remains to be explored, the enrichment analysis of SIPs highlighted functions such as cytoskeletal motor activity and microtubule motor activity, which are all needed for flagellar movements [62]. Additionally, some SIPs were associated with the GO terms “sperm binding to zona pellucida,” “prevention of polyspermy,” and “fertilization,” for which OVGP1 and MYH9 were most frequently cited. MYH9 was the most abundant SIP, and OVGP1 was among the top 10 in all conditions. OVGP1 has been shown to interact with spermatozoa in bovine [18], humans [63], and hamsters [64]. It has been previously identified as a promoter of sperm viability, motility, and capacitation in buffaloes [41]. Moreover, incubation of human spermatozoa with recombinant OVGP1 enhanced sperm capacitation [65] and binding to the oocyte [66].

Moreover, non-muscle myosins have well-documented roles in cell migration, adhesion, and movement [67–70]. In particular, MYH9 was shown to interact with the calcium-dependent S100 family member S100A4 to promote the motility of human cancer cells [67]. Of note, several S100 proteins (S100A11, S100A2, S100A14, S100B) were found in the proteome of bull sperm in the present study. A recent study reported cytoskeletal abnormalities of Sertoli cells and infertility in male mice with mutations in MYH9 [68]. Moreover, MYH9 has well-documented roles in cell exocytosis [71, 72], a process essential for sperm acrosome reaction and fertilization [73]. The numerous myosins identified as SIPs in this study (MYH9, MYH10, MYH14, MYH11, MYL6, MYL9, MYO6, MYO18A, MYO1C) open up new exciting research perspectives on sperm capacitation.

### Limitations of the study

Post-mortem collection of OF was chosen to avoid contamination by inflammatory cells and hemoglobin and for ethical and economic reasons. Though oviducts were immediately placed on ice after collection, some minor modifications in OF protein content cannot be excluded.

OF protein concentration was set at 3 mg/mL to detect sperm-interacting proteins according to preliminary data on MYH9, a known SIP, using immunoblotting. However, this concentration may not be adequate for the identification of all potential SIPs, which have various initial abundance in the OF and may have different binding affinity on sperm.

The same pool of semen from three bulls was used in this study in order to focus of female factors regulating SIPs. However, sperm-OF interactions may vary from one bull to another. Furthermore, frozen-thawed spermatozoa were used in this study for practical reasons and because artificial insemination is typically made with frozen semen in cattle. However, the cryopreservation procedure induces sperm surface modifications [74] which may modify protein interactions. Furthermore, a short incubation time (60 min) was chosen because it was sufficient to allow sperm protein interactions without affecting sperm membrane integrity and motility. However, in vivo, mammalian spermatozoa may stay up to several days in the oviduct until fertilization [75]. Longer incubation times would probably enable more and/or different OF proteins to interact with spermatozoa.

## Conclusion

To conclude, this study provides new information on OF proteins interacting with bull spermatozoa around the time of ovulation. Some of these proteins fluctuated in number and abundance, suggesting rapid changes in in vivo sperm protein surfaces, possibly to promote sperm capacitation and fertilization in the oviduct. Further work is now needed to assess the effect of recombinant SIPs on sperm, with potential applications in media used for sperm conservation, insemination, and in vitro fertilization.

## Supplementary Information

**Additional file 1: Table S1. **List of antibodies used for immunoblotting.

**Additional file 2: Table S2. **List of proteins identified and quantified in oviduct fluid (OF) from ampulla and isthmus at pre-ovulatory (Pre-ov) and post-ovulatory (Post-ov) stages and in OF-treated and control bull spermatozoa.

**Additional file 3: Table S3. **List of all sperm-interacting proteins and prediction of conventional and unconventional secretion pathways by Outcyte and SignalP and previous reports in oviduct extracellular vesicles.

**Additional file 4: Table S4. **List of sperm-interacting proteins quantified in the pre-ovulatory and post-ovulatoryampulla and isthmus with normalized quantitative values and fold-change ratios betweentreated and control sperm.

**Additional file 5: Table S5. **Functional enrichment analysis of sperm-interacting proteins for Gene Ontology (GO) Molecular Functions (MF) and Biological Processes (BP) from Metascape.

**Additional file 6: Table S6. **Protein-protein interaction network from STRING of all sperm-interacting proteins in each region × stage condition.

**Additional file 7: Fig. S1. **Sperm amplitude of lateral head (ALH), linearity (LIN), average path velocity (VAP), curvilinear velocity (VCL) and straight linevelocity (VSL) during the 60-min incubation with oviduct fluid (treated groups) or PBS (controls). Data are means ± SEM of 4 replicates.

**Additional file 8: Fig. S2. **Immunoblotting of HSPA5, MYH9 and PRDX6 in oviduct fluid used for sperm incubation and in sperm samples. The histograms indicate the mean levels of signals from three replicates. Letters indicated the significant level after ANOVA and Tuckey post-test between PBS (control) and OF-treated spz : b: *P*-value < 0.05;c: *P*-value < 0.01; d: *P*-value < 0.001.

**Additional file 9: Fig. S3. **Scatter plots of the abundance of sperm-interacting proteins (SIPs) intreated spermatozoa according to the initial abundance in oviduct fluid.

**Additional file 10: Fig. S4. **Protein-proteininteraction network of sperm-interacting proteins identified in the (A) post-ovulatoryisthmus, (B) pre-ovulatory ampulla, and (C) post-ovulatory ampulla.

## Data Availability

The datasets supporting the conclusions of this article are available in the ProteomeXchange Consortium via the PRIDE (Perez-Riverol et al., 2022) repository (www.ebi.ac.uk/pride/archive/login). Username: reviewer_pxd035271@ebi.ac.uk; Password: oFb3r6W5.

## Abbreviations

Amp Post: Post-ovulatory Ampulla
Amp Pre: Pre-ovulatory Ampulla
ANX: Annexin
BP: Biological Process
EVs: Extracellular Vesicles
FDR: False Discovery Rate
GO: Gene Ontology
GRP78: Glucose Regulated Protein 78
HSP: Heat Shock Protein
Isth Post: Post-ovulatory Isthmus
Isth Pre: Pre-ovulatory Isthmus
MF: Molecular function
MYH: Myosin
NanoLC-MS/MS: Nano Liquid Chromatography Coupled with Tandem Mass Spectrometry
NWS: Normalized Weighted Spectra
OVGP1: Oviductin
PBS: Phosphate-buffered saline
Post-ov: Post-ovulatory
PRDX6: Peroxiredoxin-6
Pre-ov: Pre-ovulatory
SDS: Sodium Dodecyl Sulfate
SIPs: Sperm-Interacting Proteins
Spz: Spermatozoa
TPM3: Tropomyosin 3
Unconv: Unconventionnal Secretion Pathways
