# Supplementary figures and images for: The sperm-interacting proteome in the bovine isthmus and ampulla during the periovulatory period
Source: J Anim Sci Biotechnol. 2023 Feb 17;14:30. doi: 10.1186/s40104-022-00811-2 (PMC9936689; doi:10.1186/s40104-022-00811-2)

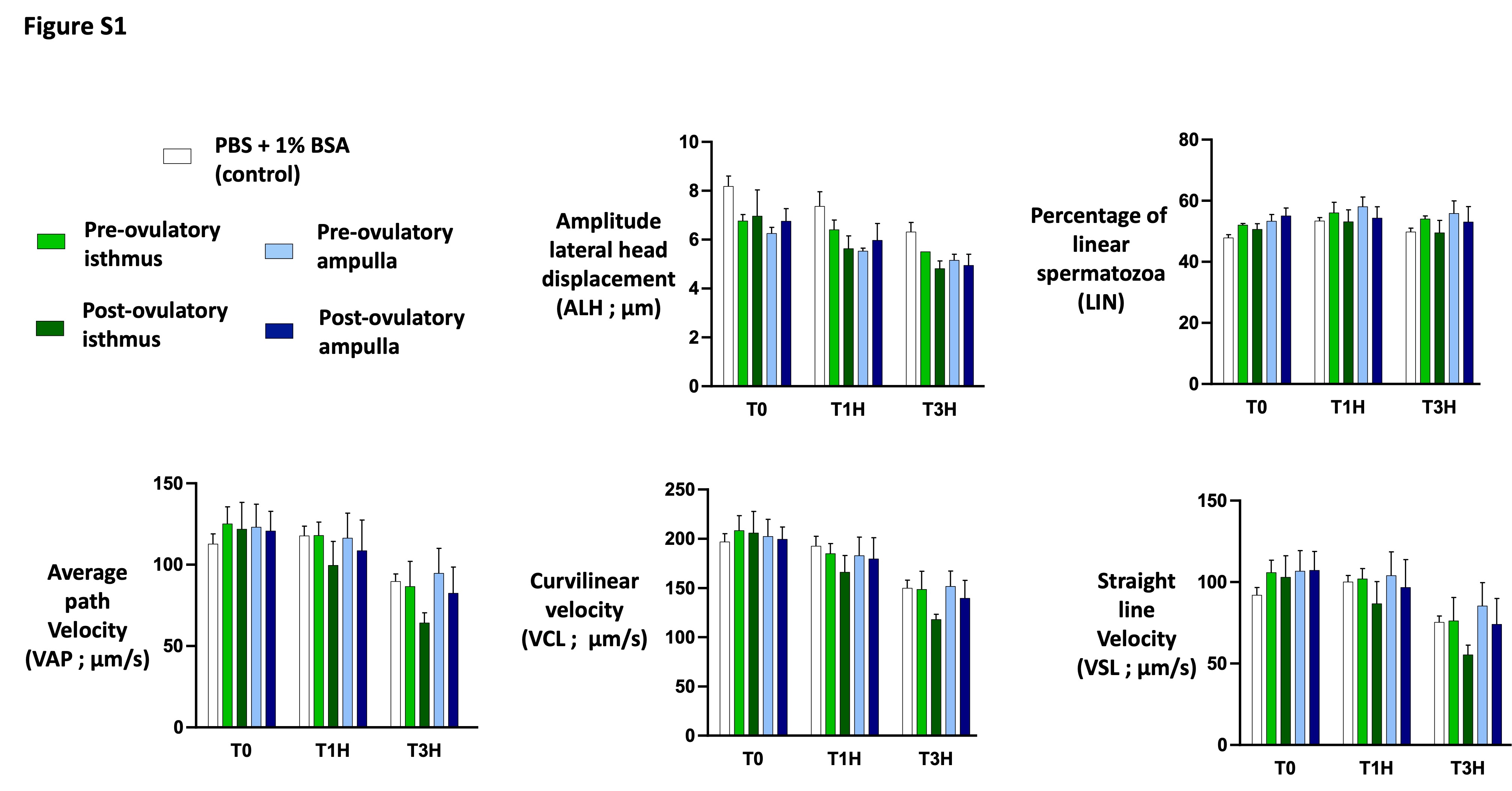

Supplement: Supplementary file 7 — Additional file 7: Fig. S1. Sperm amplitude of lateral head (ALH), linearity (LIN), average path velocity (VAP), curvilinear velocity (VCL) and straight linevelocity (VSL) during the 60-min incubation with oviduct fluid (treated groups) or PBS (controls). Data are means ± SEM of 4 replicates. [file 40104_2022_811_MOESM7_ESM.jpg]

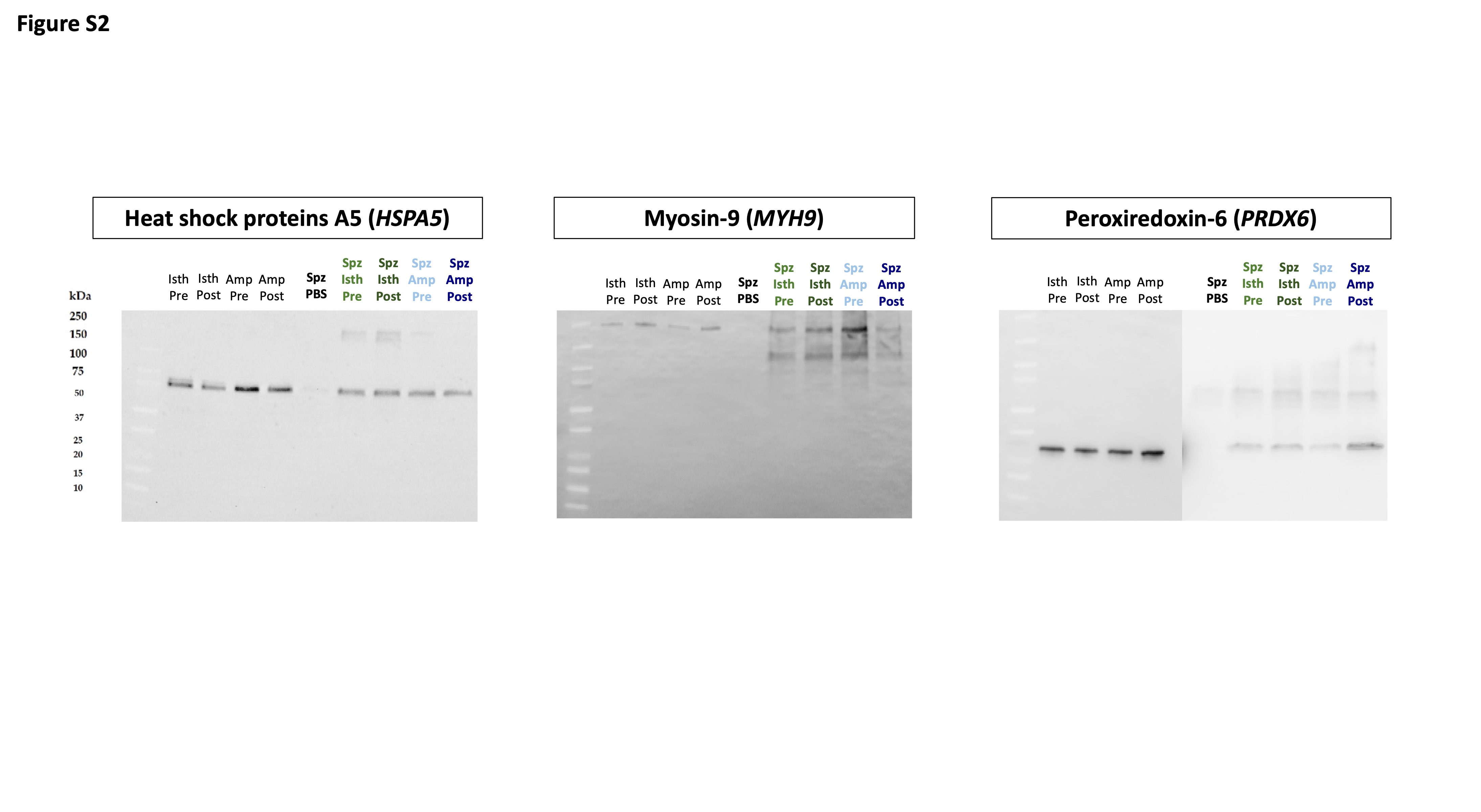

Supplement: Supplementary file 8 — Additional file 8: Fig. S2. Immunoblotting of HSPA5, MYH9 and PRDX6 in oviduct fluid used for sperm incubation and in sperm samples. The histograms indicate the mean levels of signals from three replicates. Letters indicated the significant level after ANOVA and Tuckey post-test between PBS (control) and OF-treated spz : b: P-value < 0.05;c: P-value < 0.01; d: P-value < 0.001. [file 40104_2022_811_MOESM8_ESM.jpg]

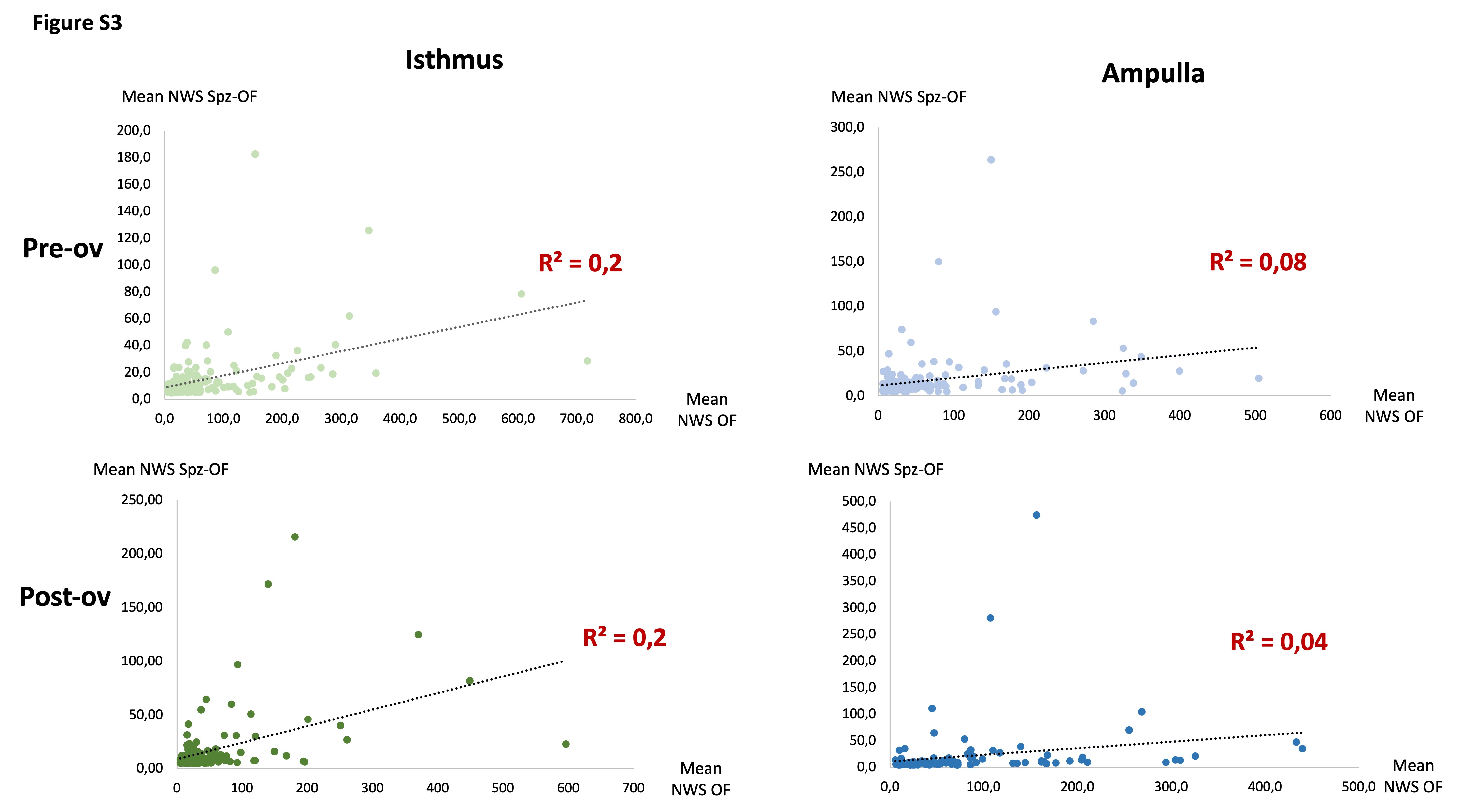

Supplement: Supplementary file 9 — Additional file 9: Fig. S3. Scatter plots of the abundance of sperm-interacting proteins (SIPs) intreated spermatozoa according to the initial abundance in oviduct fluid. [file 40104_2022_811_MOESM9_ESM.jpg]

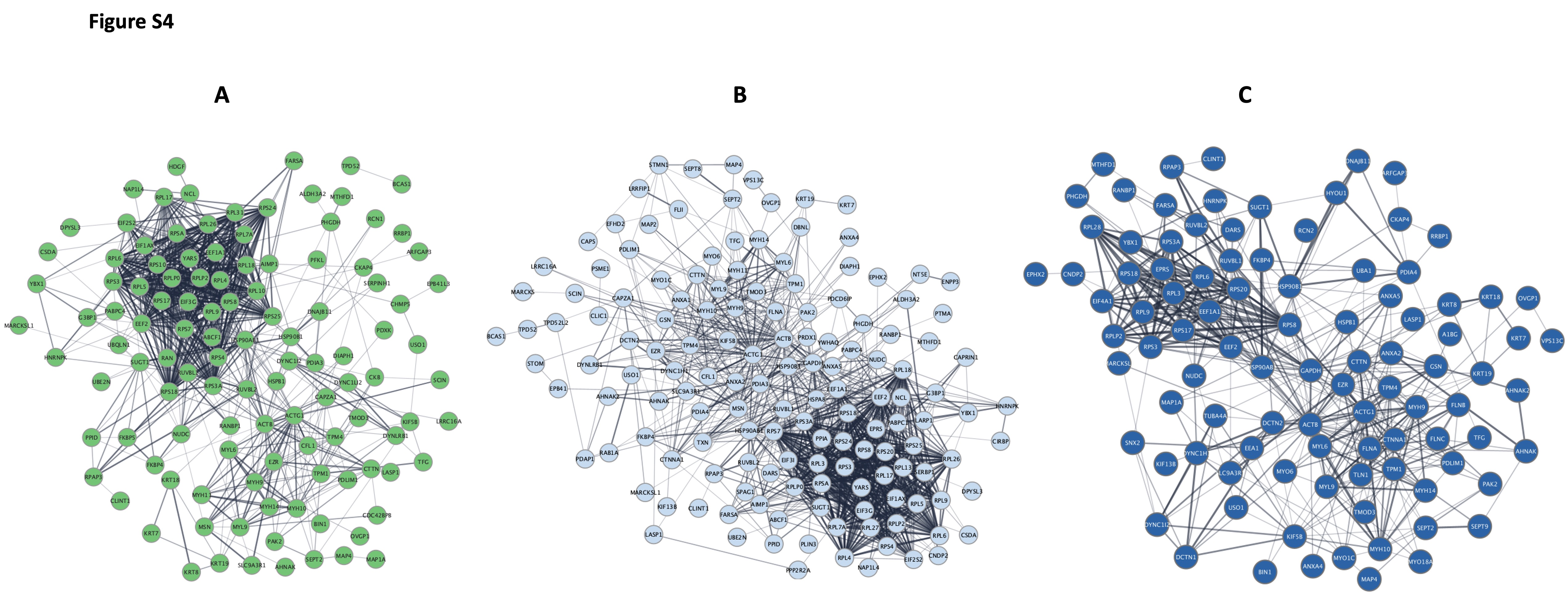

Supplement: Supplementary file 10 — Additional file 10: Fig. S4. Protein-proteininteraction network of sperm-interacting proteins identified in the (A) post-ovulatoryisthmus, (B) pre-ovulatory ampulla, and (C) post-ovulatory ampulla. [file 40104_2022_811_MOESM10_ESM.jpg]
